# Short Fetal Leukocyte Telomere Length and Preterm Prelabor Rupture of the Membranes

**DOI:** 10.1371/journal.pone.0031136

**Published:** 2012-02-13

**Authors:** Ramkumar Menon, Jie Yu, Patrice Basanta-Henry, Lina Brou, Sarah L. Berga, Stephen J. Fortunato, Robert N. Taylor

**Affiliations:** 1 Division of Maternal-Fetal Medicine and Perinatal Research, Department of Obstetrics and Gynecology, The University of Texas Medical Branch at Galveston, Galveston, Texas, United States of America; 2 Department of Gynecology and Obstetrics, Emory University, Atlanta, Georgia, United States of America; 3 Department of Obstetrics and Gynecology, Wake Forest School of Medicine, Winston-Salem, North Carolina, United States of America; 4 Department of Epidemiology, Emory University, Atlanta, Georgia, United States of America; 5 The Perinatal Research Center, Nashville, Tennessee, United States of America; University of Florida, United States of America

## Abstract

**Background:**

Rupture of the fetal membranes is a common harbinger of imminent labor and delivery. Telomere shortening is a surrogate for oxidative stress (OS) and senescence. Fetal leukocyte and placental membrane DNA telomere lengths were evaluated to determine their association with preterm prelabor rupture of the membranes (pPROM) or spontaneous preterm births with intact membranes (PTB), compared to term birth.

**Methods:**

Telomere lengths were quantified in cord blood leukocytes (n = 133) from three major groups: 1) pPROM (n = 28), 2) PTB (n = 69) and 3) uncomplicated full term births (controls, n = 35), using real-time quantitative PCR. Placental membrane specimens (n = 18) were used to correlate fetal leukocyte and placental telomere lengths. Telomere length differences among the groups were analyzed by ANOVA. Pearson correlation coefficients determined relationships between leukocyte and placental membrane telomere lengths.

**Results:**

In pregnancies with intact membranes, fetal leukocyte telomere length was inversely proportional to gestational age. The mean telomere length decreased as gestation progressed, with the shortest at term. pPROM had telomere lengths (9962±3124 bp) that were significantly shorter than gestational age-matched PTB (11546±4348 bp, p = 0.04), but comparable to term births (9011±2497 bp, p = 0.31). Secondary analyses revealed no effects of race (African American vs. Caucasian) or intraamniotic infection on telomere length. A strong Pearson's correlation was noted between fetal leukocyte and placental membrane telomere lengths (ρ = 0.77; p<0.01).

**Conclusions:**

Fetal leukocyte telomere length is reduced in pPROM compared to PTB but is similar to term births. pPROM represents a placental membrane disease likely mediated by OS-induced senescence.

## Introduction

Preterm (<37 weeks of completed gestation) prelabor rupture of the membranes (pPROM) occurs in about 3–4% of all pregnancies. pPROM is directly antecedent to 40% to 50% of all preterm births and occurs in many women without identifiable risk factors [1]. Despite remarkable improvements in prenatal care over the past three decades, rates of pPROM and subsequent preterm delivery have worsened [2]. While several tests are available to confirm pPROM post facto (e.g. amniotic fluid pooling, “ferning”, nitrazine reaction, and Amnisure®), no method exists to reliably predict pPROM [3], [4]. This dilemma is mostly attributable to the fact that precise risk factors, causes, or pathways resulting in pPROM are unknown. Proper diagnosis and management of pPROM is likely to require thorough investigation of specific exposure-induced pathophysiologic pathways and the development of corresponding biomolecular markers. Several epidemiological and clinical factors are considered precursors to pPROM [3], [4], [5], including maternal reproductive tract infections (e.g., bacterial vaginosis [BV], trichomoniasis, gonorrhea, Chlamydia and occult chorioamnionitis), behavioral factors (e.g., cigarette smoking, substance abuse, poor nutritional status, and coitus during pregnancy), obstetric complications (e.g., multiple gestation, polyhydramnios, incompetent cervix, uterine bleeding), prior cervical surgery, and antenatal trauma. Environmental factors (e.g., stress, toxin exposure) and genetic predisposition also have been proposed. In addition, biochemical signals from the fetus, including endocrine signals that promote placental membrane apoptosis, have been implicated in pPROM [3], [4], [5], [6], [7], [8], [9], [10].

Recent histologic and biomarker analyses from our laboratory and others' suggest common placental membrane changes in pPROM ending in preterm birth and normal term birth. At term, oxidative stress (OS) and senescence are associated with placental membrane apoptosis and collagenolysis (required for membrane degradation, rupture and cervical ripening), which contribute to normal parturition [7], [10]. In contrast to pregnancies associated with preterm birth with intact membranes (PTB), pPROM and term pregnancies are characterized by the following features: 1) placental membrane apoptosis or necrosis [8]; 2) elevated amniotic fluid (AF) inflammatory markers [6]; 3) high salivary (collagenolytic activity a surrogate for protease activation in the lower uterine segment) [9]; and 4) elevated AF F2-IsoP concentrations (a biomarker of oxidative stress [OS]) [11]. Except for elevated inflammatory markers (interleukins and chemokines), these factors differ between pPROM and gestational age-matched PTB with intact membranes. These findings led us to hypothesize that pPROM is a disease of the placental membranes wherein multiple risk factors associated with OS and inflammation accelerate membrane senescence, apoptosis and proteolysis, resulting in pPROM. In this study, we quantified fetal leukocyte telomere length as a marker of OS and cellular aging [12],[13]. Telomeres are DNA-protein complexes that cap the ends and preserve chromosomal stability throughout the cell cycle [14], [15]. When chromosomes undergo replication during cell division, the telomere is not fully replicated secondary to limitations of DNA polymerase activity at the 5′ end of the lagging strand, resulting in progressive telomere shortening with each cell cycle. Thus, telomere lengths serve as a valid marker of a cell's “biologic age” [13], [14]. Emerging studies suggest that in adults, biochemical mediators of physiological and psychological stress result in OS, accelerate telomere shortening and advance cellular aging [15], [16].Cellular senescence, as evidenced by shortened telomeres, is a predictor of mortality [16] and a number of chronic diseases such as obesity, diabetes, cardiovascular and inflammatory disorders [17], [18], [19].

Our study analyzed fetal leukocyte telomere length during gestation, comparing telomere length among products of pPROM, PTB, and normal term births to ascertain the risk of the three pregnancy outcomes in subjects with reduced telomere length. Racial disparity is associated with pPROM and preterm birth rates and we have provided evidence that genetic and inflammatory markers may contribute to this phenomenon [19]. Therefore, secondary analyses were performed to investigate if racial disparity or intraamniotic infection affected telomere length.

## Methods

This study was conducted at Centennial Women's Hospital, Nashville, TN and Emory University, Atlanta, GA and was approved by the TriStar Nashville institutional review board (Centennial Medical Center), Western Institutional Review Board (Seattle, WA) and the Emory University IRB (Atlanta, GA). Subjects were recruited at Centennial Women's Hospital between September 2006 and December 2010 for studies of genetics and biomarkers associated with PTB as reported previously [20]. All subjects enrolled in this study provided written consent to participate in this investigation.

Mothers between the ages of 18 and 40 were recruited. Gestational age was determined by last menstrual period and corroborated by ultrasound dating. pPROM was defined as rupture of membranes confirmed by tests such as AF pooling, “ferning”, nitrazine, and Amnisure positivity followed by delivery prior to 37^0/7^ weeks' gestation. PTB was defined as presence of regular uterine contractions at ≤37^0/7^weeks' gestation (2 contractions/10 minutes with documented cervical change) followed by delivery. Subjects with multiple gestations, preeclampsia, placental previa, infant anomalies, gestational diabetes, poly- or oligohydramnios, and other complications including surgery during pregnancy were excluded. Controls were women having normal labor and delivery at term (≥37^0/7^ weeks) without medical or obstetrical complications and no prior history of pPROM or PTB. Women who were treated for preterm labor and delivered at term were also excluded.

In this study pPROM is considered the “case” group and PTB with intact membranes and normal term births were used as “controls” for statistical comparisons.

### Identification of race

Race was identified by self-report using a questionnaire that traces ethnicity back two generations from the parents. Individuals who reported more than one racial group in their ancestry were excluded from the study [20].

### Fetal cord blood collection, DNA sampling and genotyping

Fetal cord blood samples were collected in EDTA tubes immediately after delivery and tubes were filled to capacity to minimize ex vivo oxidation effects. From a random subgroup of subjects, placental membrane specimens were collected (pPROM = 5, PTB = 5, and term = 8) to correlate placental and fetal leukocyte telomere lengths. Reflected membranes were separated from the placenta after delivery and mid-zone tissues (away from the cervix) were excised, and washed in Hank's Balanced Salt Solution (Sigma, St. Louis, MO). Samples were stored in ALLProtect® tissue reagent (Qiagen) for future DNA/RNA and protein extraction. Leukocyte DNA was isolated from fetal blood samples using the Autopure automated system (Gentra Systems, Minneapolis, MN). Placental membrane DNA was extracted using Trizol (Invitrogen, Carlsbad, CA).

### Telomere length measurement by real time quantitative polymerase chain reaction (qPCR)

Telomere length was determined in fetal cord blood leukocytes and placental membrane genomic DNA samples using real time qPCR [21]. Our pilot studies indicated that to detect a difference of 300 bp, with α = 0.05 and β = 0.80, 28 samples in each group would be adequate. Duplicate PCR reactions were run for each sample using 5 µL of each DNA dilution. A PCR mix of 15 µL containing reagents and primers for telomere length determinations and measurement of the single copy gene 36B4 (control reaction) was added to each DNA solution for a final 20 µL volume as described [21], [22]. Reactions were carried out using a BioRad Opticon2 DNA Engine and software (Hercules, CA). The primer sequences were as described by Gil and Coetzer [22]: telomere (sense), 5′-CGGTTTGTTTGGGTTTGGGTTTGGGTTTGGGTTTGGGTT-3′; telomere (anti-sense), 5′-GGCTTGCCTTACCCTTACCCTTACCCTTACCCTTACCCT-3′; 36B4 (sense), 5′-CAGCAAGTGGGAAGGTGTAATCC-3′; 36B4 (anti-sense), 5′-CCCATTCTATCATCAACGGGTACAA-3′. The qPCR program used the following parameters: 95°C×15 min for initial denaturation, 95°C×5 sec, 56°C×30 sec and 72°C×60 sec with 40 cycles of amplification. The single copy 36B4 gene product was amplified as follows: 95°C×15 min for initial denaturation, 95°C×5 sec, 58°C×30 sec and 72°C×60 sec with 40 cycles of amplification. Telomere length was calculated using the empirically derived quadratic formula: telomere length (bp) = 37631x^2^−85075x+53005. Reference standards of genomic DNA with known telomere length (from pooled “old”, “middle-aged” and “young” subjects) were used to normalize the assay.

### Statistical analysis

Data are presented as means ± SD. For continuous and categorical variables, respectively, Student's *t*-tests and χ^2^ tests were performed to determine the statistical significance of any differences in the distribution of demographic and clinical characteristics among pPROM, PTB and term births (pPROM vs. term; PTB vs. term; pPROM vs. PTB). Statistical comparisons were performed using one-way ANOVA with Bonferroni corrections to determine if telomere length differed among pPROM, PTB, and term births. Pearson correlation coefficients were calculated to determine if gestational age and telomere length correlate in pPROM, PTB, and term births and also to document the relationship between fetal leukocyte and placental membrane telomere lengths. Due to the categorical nature of the outcome, polytomous logistic regression models were used to estimate odds ratios (OR) and 95% confidence intervals (CI) for pPROM birth compared to PTB, and pPROM compared to term birth in bivariate and multivariate models. Potential confounding factors included maternal smoking during pregnancy and marital status. All analyses were performed in SAS 9.2 (SAS Institute Inc, Cary, NC).

## Results

A total of 132 fetal leukocyte DNA samples were analyzed in this study, which included 28 pPROM, 69 PTB with intact membranes, and 35 normal term births. Demographic details of the subjects are provided in Tables 1 and 2. No significant differences were noted among pPROM, PTB, and term birth groups with respect to maternal age, BMI, education, employment, income, or insurance status. Birth weight, Apgar score (1 minute), and birth latency differed significantly between cases (pPROM and PTB) and term deliveries. By definition, gestational ages also were significantly greater in termbirths. Compared to PTB, the pPROM group had disproportionately more single mothers, cigarette smokers, and cases with histologic chorioamnionitis (p<0.01).

**Table 1:** Demographic characteristics of subjects included in this study.

Demographics	pPROM^1^ (n = 28)	Two-tailed p-values^4^	Preterm^1^ (n = 69)	Two-tailed p-values^3^	Term^1^ (n = 35)	Two-tailed p-values^2^
**Marital Status, n (%)**						
Single or Divorced	19 (70.4)	0.053	27 (41.5)	0.012	14 (45.2)	0.737
Married	8 (29.6)		38 (58.5)		17 (54.8)	
**Smoking, n (%)**						
No	26 (92.9)	0.49	49 (71.0)	0.02	28 (87.5)	0.07
Yes	2 (7.1)		20 (29.0)		4 (12.5)	
**Gestational Age, days, mean (SD)**	210.3 (24.2)	<0.0001	213.9 (37.4)	0.635	268.1 (20.1)	<0.0001
**Birthweight, g, mean (SD)**	1669.3 (701.4)	<0.0001	1833.2 (909)	0.395	2859.5 (1009.2)	<0.0001
**Apgar Score, mean (SD)**						
At 1 min	6.8 (2.2)	0.074	6.8 (2.1)	0.99	7.6 (1.6)	0.038
At 5 min	8.0 (1.3)	0.13	8.1 (1.3)	0.64	8.5 (1.5)	0.163
**Race, n (%)**						
African American	13 (46.4)	0.866	37 (53.6)	0.521	17 (48.6)	0.626
Caucasian	15 (53.6)		32 (46.4)		18 (51.4)	
**Latency of Preterm Labor, mean (SD)**	8.4 (13.6)	0.014	9.4 (15.1)	0.76	2.7 (3.9)	0.001
**Latency of pPROM, mean (SD)**	9.2 (9.9)	0.017	0.17 (0.4)	0.02		

**Note: Missing data in some variables.**

**Table 2:** Data on clinical variables in pPROM and PTB groups.

Clinical Variables	pPROM^1^	Preterm^1^	Two-tailed p-value*
	(n = 28)	(n = 69)	
*Infection*			
No	15 (53·6)	53 (80·3)	0·008
Yes	13 (46·4)	13 (19·7)	
*Group B Strep*			
No	16 (69·6)	35 (74·5)	0·665
Yes	7 (30·4)	12 (25·5)	
*Clinical Chorioamnionitis*			
No	11 (40·7)	36 (59·0)	0·113
Yes	16 (59·3)	25 (41·0)	
*Histological Chorioamnionitis*			
No	14 (50·0)	52 (77·6)	0·008
Yes	14 (50·0)	15 (22·4)	
*Funisitis*			
No	21 (77·8)	60 (88·2)	0·195
Yes	6 (22·2)	8 (11·8)	
Yes	18 (65.0)	26 (39·4)	

*Based on Student's t-test.

Initially, we calculated fetal leukocyte telomere length in pregnancies delivered with intact membranes. Telomere length was normally distributed (Shapiro-Wilks p-value = 0.15), met the other assumptions of ANOVA (linearity, homoscedasticity, and independence) and was not found to be collinear among groups (condition index<10). As anticipated, we observed a general trend that telomere length was inversely proportional to gestational age. The longest telomeres (25258 bp) were noted in a PTB sample at 174 days gestation and the shortest telomeres (5022 bp) in a term pregnancy delivered at 282 days. These data should be interpreted with caution as fetal telomeres from PTB may be confounded by pathological complications leading to early delivery.

Primary analyses compared telomere lengths among pPROM, PTB, and term birth (Table 3). Overall, telomere length was not different between pPROM (9962±3124 bp) and term birth (9011±2497 bp; p = 0.31). PTB had significantly longer telomeres (11546±4348 bp) than either pPROM (p = 0.05) or term birth (p<0.01) groups. When the data were further stratified based on gestational age, early pPROM and PTB (≤32 weeks) had similar evidence of infection, inflammation and low birth weight. Newborns from PTB≤32 weeks had longer telomeres (11679±4926 bp) than those of gestation age-matched pPROM (9432±2618 bp; p = 0.01), whereas no difference was seen in telomere length between fetuses from pPROM (12400±4374 bp) and PTB>32 weeks (11416±3771 bp; p = 0.63). Interestingly, pPROM≤32 weeks had similar fetal telomere length (9432±2618 bp) to that detected in term births (9011±2497 bp; p = 0.67). However, marginally longer telomeres were observed in pPROM≥32 weeks (12400±4374 bp) compared to term births (9011±2497 bp; p = 0.07).

**Table 3:** Telomere length comparison between pPROM, PTB and term birth.

Telomere Length	N	Mean ± SD	p-value^1^	p-value^2^
pPROM	28	9962·3±3124·1	0·31	0·05
pPROM - <32 weeks	23	9432·4±2618·2	0·67	0.01
pPROM - >32 weeks	5	12400·1±4374·2	0·07	0·63
Preterm Birth	69	11546·4±4348·2	0·001	-
Preterm Birth - <32 weeks	35	11679·6±4926·3	0·003	-
Preterm Birth - >32 weeks	34	11416·9±3771·6	0·002	-
Term Birth	35	9011·1±2497·3	-	-

1 ANOVA p-values with Bonferroni correction of comparison with term controls.

2 ANOVA p-values with Bonferroni correction of comparison with preterm cases.

Secondary analyses were performed to ascertain whether fetal leukocyte telomere length related to race or intraamniotic infection (IAI) status (documented by microbial culture of amniotic fluid, histologic chorioamnionitis or funisitis, or by clinical signs of infection that included fever [>102°F], high C-reactive protein, abdominal tenderness or foul smelling vaginal discharge). Racial disparity was not observed in fetal telomere length among any of the three conditions (pPROM, PTB or term birth). Regardless of infection status, pPROM was associated with shorter telomere lengths (IAI, 9949±3496 bp, no IAI, 9973±2888 bp) than gestational age-matched PTB (IAI, 12859±5373 bp, no IAI, 11395±4114 bp); however, these data failed to reach statistical significance (IAI, p = 0.12; no IAI, p = 0.22).

We derived a model to calculate the risk of pPROM, based on telomere length. Our data would predict that a 1000 bp decrease in telomere length would increase the risk of pPROM by 1.12 (95% CI, 0.99–1.38; p = 0.08) compared to PTB with intact membranes. When the analysis was restricted to early pPROM (≤32 weeks of gestational age), the OR (95% CI) was 1.26 (1.02–1.38) for a 1000 bp decrease in telomere length compared to PTB (p = 0.03). As expected, fetal telomere length was not a predictor of pPROM when compared to term births (p = 0.27). Although a significant result was indicated in bivariate analysis for PTB compared to pPROM, statistical significance was not achieved in multivariate analysis (OR (95% CI) = 1.11 (0.94–1.30); p = 0.220).

Analyses were performed to correlate matched fetal leukocyte and placental membrane telomere lengths. Telomere lengths in placental membranes were systematically longer than corresponding fetal leukocyte telomeres; however, the same trends in pPROM and PTB were maintained. Placental membrane telomeres were shorter in pPROM (9227±3389 bp) compared to PTB (14444±3320 bp; p = 0.05), whereas no differences were seen between pPROM and term birth (11842±4456 bp; p = 0.32). A strong Pearson's correlation was observed between placental membrane and fetal DNA telomere lengths (ρ = 0.77; p<0.01) despite a small sample size.

## Discussion

It remains unclear why some premature births present with pPROM whereas others have spontaneous PTB with intact membranes. Both pPROM and PTB share similar etiologic and pathophysiologic pathways, especially at early gestational ages (≤32 weeks) [1], [4]. Infection with prominent cytokine and chemokine responses is a major factor associated with both conditions [23]. Conversely, placental membrane apoptosis and MMP activation are more common in pPROM than PTB [3], [5], [7], [8],[10], leading to membrane weakening and rupture in the former cases. Recent findings from our laboratory indicate that cigarette smoking induces OS and apoptotic cell death in placental membranes, even in the absence of inflammation, predisposing them to rupture [11], [24]. Higher F2-Isoprostane (F2-IsoP, a marker of OS) levels in the amniotic fluid of women who smoked during pregnancy confirm these findings [24]. Similarly, in a pilot project, we were able to document that AF F2-IsoP concentrations were higher in pPROM subjects than gestational age-matched PTB with intact membranes (2.64±4.29 ng/ml vs. 0.31±0.31 ng/ml; p<0.01, unpublished data) further supporting the role of OS during pPROM. These data prompted us to investigate whether cellular senescence of the placental membranes associated with pPROM was likely due to OS.

Inflammation and particularly OS can promote telomere shortening. Telomeres are complexes of proteins (including telomerase reverse transcriptase [hTERT] and dyskerin [DKC1]) and short, noncoding RNAs that add DNA sequence repeats (“TTAGGG”) to the 3′ ends of replicating DNA strands to maintain structural and functional integrity of the distal chromosomes [14], . During each round of replication, human telomeres lose 20–100 bp of repeated sequence. After ∼60 cell divisions, as predicted by Hayflick [26], human telomeres become short, triggering apoptosis [27]. Our identification of short fetoplacental telomeres at term is consistent with the negative correlation of telomere length with physiologic cellular aging during gestation.

We tested telomere length differences in pPROM compared to gestational age-matched PTB with intact membranes and normal term births. Telomere length serves as a surrogate for fetal stress, placental membrane senescence and as a marker for OS. The key findings from our study are: 1) Fetal leukocyte and placental membrane telomere length is reduced as gestation progresses, with the shortest telomeres seen at term and longest seen in early PTB (25 weeks) with intact membranes; 2) Fetal telomere lengths in early pPROM (≤32 weeks) mimic those of term births, suggesting that OS and cellular aging are accelerated in pPROM; 3) PTB with intact membranesis associated with the longest telomeres; 4) Differences in fetal leukocyte telomere length among cases of early pPROM and PTB suggest that distinct pathophysiologic processes differentiate these outcomes; 5) Fetal leukocyte telomere length correlates with placental membrane telomere length in all three groups, indicating similar cellular aging processes in both compartments of the fetoplacental unit.

The responses of telomeres to reactive oxygen species (ROS) support our hypothesis and prior observations [11], [24] that specific subsets of preterm births may be caused by OS. Due to their high content of guanines, which undergo oxidative modification to 8-oxo-dG bases, telomeres are highly sensitive to damage by OS. Senescent cells have four times the 8-oxo-dG content of healthy cells [28], [29]. Furthermore, ROS, especially hydroxyl radicals, can introduce single strand DNA breaks, which are less amenable to repair in telomeric than genomic DNA. In vitro studies also indicate that infection, inflammation and inflammatory cytokines per se (e.g., TNF-α) downregulate hTERT enzyme activity, accelerating cell senescence [30]. We postulate that OS mediated telomere shortening is a normal physiologic process leading to term delivery, but is activated prematurely in pPROM.

Our study supports the hypothesis that pPROM is a disorder of the placental membranes wherein shortened fetal telomere lengths reflect precocious cellular senescence. Along with our prior reports of amniochorion apoptosis and proteolysis, these findings suggest that OS during pregnancy has selective effects on placental membrane integrity. To substantiate this theory, we also examined maternal telomere lengths from pPROM, PTB and term births. As predicted, maternal telomere lengths were shorter than those of their fetuses, but their mean lengths (8699±1849 bp in pPROM, 10467±9971 bp in PTB, and 12893±11449 bp at term) did not differ significantly with respect to delivery outcome (ANOVA, p = 0.36, preterm vs. term; p = 0.45, pPROM vs. term; p = 0.75, preterm vs. pPROM). This finding suggests that OS preferentially activates an intrauterine pathway associated with pPROM. The high correlation between fetal and placental membrane telomere lengths, but not maternal telomere lengths, indicates that the fetal and maternal compartments respond differentially to OS in pregnancy. Based on the report of Okuda et al. [31], in which telomere lengths were similar among different tissues within the same newborns, we predicted a high correlation between fetal leukocyte and placental membrane telomere lengths. While these were highly correlated (ρ = 0.77; p<0.01), placental membrane telomeres were approximately 25% longer than those in corresponding leukocytes. Data presented here suggest that fetal telomere lengths are affected by risk factors that are associated with pPROM. These factors include, but are not limited to, psychosocial and socio-economic stressors, behavioral factors (cigarette smoking, substance abuse), nutritional deficiencies, antioxidant stores, infection or inflammatory responses. Each of these factors can promote imbalances in the redox status, resulting in OS. Additionally, fetal responses to a hostile intrauterine environment may also promote OS. Although our secondary stratified analyses did not reveal differences in telomere lengths between cases with and without intraamniotic infection, this study was not powered to accurately address those questions.

We believe that this is the first report of telomere length differences among pPROM, PTB and term births. The findings may be particularly relevant in the context of fetal programming of adult diseases. Accelerated cellular aging in the fetus may portend defective telomere maintenance throughout the life of that individual, potentially impacting a variety of adult metabolic conditions. Ongoing research in our laboratory that aims to understand determinants of telomere shortening during intrauterine life may provide valuable insights into pregnancy complications and newborn and adult diseases.
